# Supplementary material for: Atypical early neural responses to native and non-native language in infants at high likelihood for developing autism
Source: Mol Autism. 2025 Feb 3;16:6. doi: 10.1186/s13229-025-00640-w (PMC11792659; doi:10.1186/s13229-025-00640-w)
Supplement: Supplementary file 1 — Supplementary Material 1 [file 13229_2025_640_MOESM1_ESM.docx]

**Supplementary Information**

| **Cluster** | **Predictor** | **F value** | **p-value** |
| --- | --- | --- | --- |
| HL < TL, English | Group | 10.22 | **0.003*** |
|  | Sex | 0.31 | 0.58 |
|  | MatEd | 2.42 | 0.13 |
|  | Birth Order | 0.35 | 0.56 |
|  | Group*Sex | 0.64 | 0.43 |
|  | Group*MatEd | 2.08 | 0.16 |
|  | Group*BirthOrder | -- | -- |
| HL < TL, Japanese brain clusters | Group | 29.58 | **<0.001 *** |
|  | Sex | 1.85 | 0.18 |
|  | MatEd | 2.25 | 0.14 |
|  | Birth Order | 8.97 | **0.005*** |
|  | Group*Sex | 1.51 | 0.23 |
|  | Group*MatEd | 2.89 | 0.10 |
|  | Group*BirthOrder | -- | -- |
| HL < TL, Japanese cerebellum clusters | Group | 12.91 | **<0.001*** |
|  | Sex | 0.003 | 0.96 |
|  | MatEd | 0.51 | 0.48 |
|  | Birth Order | 5.30 | **0.03*** |
|  | Group*Sex | 3.92 | 0.055 |
|  | Group*MatEd | 0.001 | 0.98 |
|  | Group*BirthOrder | -- | -- |

**Table S1. Effects of Potential Confounds:** Parameter estimates were extracted from brain clusters that showed significant group differences. Group differences were tested for significance using ACNOVAs. All models included scanner as a nuisance predictor. Group differences were still significant across all models tested (p-values bolded). MatEd: maternal education; HL: high likelihood for autism; TL: typical likelihood for autism.

| **Cluster** | **Predictor** | **F value** | **p-value** |
| --- | --- | --- | --- |
| HL < TL, English | Group | 11.97 | **0.001*** |
|  | LangExp | 1.70 | 0.20 |
|  | Group*LangExp | 0.57 | 0.46 |
| HL < TL, Japanese brain clusters | Group | 21.50 | **<0.001*** |
|  | LangExp | 0.11 | 0.74 |
|  | Group*LangExp | 0.54 | 0.47 |
| HL < TL, Japanese cerebellum clusters | Group | 12.84 | **<0.001** |
|  | LangExp | 0.66 | 0.42 |
|  | Group*LangExp | 2.96 | 0.09 |

**Table S2. Effects of Language Exposure**: Parameter estimates were extracted from brain clusters that showed significant group differences, and the effect of second-language exposure was tested, along with the effect of a group-by-language-exposure interaction, using ANOVA. All models included scanner as a nuisance covariate. Group differences were still significant across all of the models (p-values bolded). LangExp: second language exposure, coded as a binary variable (presence of any non-English exposure ≤ 50%, versus 100% English exposure).

| **Birth Order** | | | | |
| --- | --- | --- | --- | --- |
|  | First-born | Not First-born | ***t* or *Χ*^2^** | **P-value** |
| MSEL T-scores (12 months) |  |  |  |  |
| Gross Motor | 51.2 | 49.0 | 0.28 | 0.79 |
| Fine Motor | 69.2 | 62.4 | 1.30 | 0.23 |
| Visual Reception | 61.4 | 59.1 | 0.96 | 0.35 |
| Receptive Language | 52.0 | 51.8 | 0.08 | 0.94 |
| Expressive Language | 53.0 | 52.8 | 0.03 | 0.98 |
| MSEL T-scores (36 months) |  |  |  |  |
| Gross Motor | – | – | – | – |
| Fine Motor | 56.7 | 54.0 | 0.47 | 0.65 |
| Visual Reception | 63.0 | 59.7 | 0.74 | 0.46 |
| Receptive Language | 63.7 | 52.2 | 2.66 | **0.02*** |
| Expressive Language | 63.9 | 52.7 | 3.76 | **0.001**** |
| Vineland (12 months) |  |  |  |  |
| Communication | 100.4 | 88.5 | 1.67 | 0.12 |
| Socialization | 91.0 | 83.0 | 0.53 | 0.61 |
| Vineland (36 months) |  |  |  |  |
| Communication | 110.5 | 99.13 | 3.04 | **0.005**** |
| Socialization | 108.38 | 98.06 | 2.74 | **0.01*** |
| **Sex** | | | | |
|  | Male | Female | ***t* or *Χ*^2^** | **P-value** |
| MSEL T-scores (12 months) |  |  |  |  |
| Gross Motor | 52.1 | 47.4 | 0.80 | 0.43 |
| Fine Motor | 63.3 | 65.0 | 0.37 | 0.71 |
| Visual Reception | 61.0 | 58.2 | 1.04 | 0.31 |
| Receptive Language | 50.6 | 53.7 | 1.10 | 0.28 |
| Expressive Language | 52.0 | 54.4 | 0.45 | 0.66 |
| MSEL T-scores (36 months) |  |  |  |  |
| Gross Motor | – | – | – | – |
| Fine Motor | 47.80 | 63.94 | 3.90 | **<0.001***** |
| Visual Reception | 57.21 | 64.65 | 1.65 | 0.11 |
| Receptive Language | 51.29 | 58.29 | 1.76 | 0.09 |
| Expressive Language | 51.38 | 59.24 | 2.36 | **0.02*** |
| Vineland (12 months) |  |  |  |  |
| Communication | 92.3 | 89.9 | 0.26 | 0.80 |
| Socialization | 91.8 | 76.4 | 1.02 | 0.33 |
| Vineland (36 months) |  |  |  |  |
| Communication | 95.0 | 109.2 | 3.23 | **0.003**** |
| Socialization | 91.5 | 110.6 | 4.43 | **<0.001***** |
| **Maternal Education** | | | | |
|  | Some College | Some Grad | ***t* or *Χ*^2^** | **P-value** |
| MSEL T-scores (12 months) |  |  |  |  |
| Gross Motor | 50.8 | 47.5 | 0.87 | 0.39 |
| Fine Motor | 60.4 | 65.9 | 1.97 | 0.06 |
| Visual Reception | 58.7 | 57.9 | 0.33 | 0.74 |
| Receptive Language | 48.0 | 51.0 | 1.25 | 0.22 |
| Expressive Language | 48.3 | 52.9 | 1.38 | 0.17 |
| MSEL T-scores (36 months) |  |  |  |  |
| Gross Motor | – | – | – | – |
| Fine Motor | 52.65 | 56.24 | 0.75 | 0.46 |
| Visual Reception | 56.70 | 64.88 | 1.78 | 0.08 |
| Receptive Language | 50.43 | 59.35 | 2.19 | **0.04*** |
| Expressive Language | 51.87 | 58.41 | 1.82 | 0.08 |
| Vineland (12 months) |  |  |  |  |
| Communication | 90.3 | 92.7 | 0.37 | 0.72 |
| Socialization | 91.8 | 76.4 | 1.02 | 0.33 |
| Vineland (36 months) |  |  |  |  |
| Communication | 95.73 | 108.82 | 2.87 | **0.007**** |
| Socialization | 95.32 | 107.53 | 2.53 | **0.02*** |
| **Second Language Exposure** | | | | |
|  | Monolingual | 2^nd^ Lang Exp. | ***t* or *Χ*^2^** | **P-value** |
| MSEL T-scores (12 months) |  |  |  |  |
| Gross Motor | 47.4 | 50.7 | 0.89 | 0.38 |
| Fine Motor | 61.5 | 63.5 | 0.63 | 0.53 |
| Visual Reception | 58.6 | 58.0 | 0.26 | 0.80 |
| Receptive Language | 49.5 | 49.1 | 0.18 | 0.86 |
| Expressive Language | 49.8 | 51.0 | 0.32 | 0.75 |
| MSEL T-scores (36 months) |  |  |  |  |
| Gross Motor | – | – | – | – |
| Fine Motor | 53.39 | 55.35 | 0.39 | 0.70 |
| Visual Reception | 59.17 | 61.17 | 0.39 | 0.70 |
| Receptive Language | 50.06 | 57.43 | 1.67 | 0.10 |
| Expressive Language | 50.39 | 57.96 | 2.01 | 0.05 |
| Vineland (12 months) |  |  |  |  |
| Communication | 92.8 | 90.2 | 0.39 | 0.70 |
| Socialization | 84.6 | 86.8 | 0.23 | 0.82 |
| Vineland (36 months) |  |  |  |  |
| Communication | 99.24 | 103.0 | 0.73 | 0.47 |
| Socialization | 98.12 | 101.61 | 0.65 | 0.52 |

**Table S3. Relationships between potential confound variables and language scores.** Second language exposure was coded as a binary variable (presence of any non-English exposure ≤ 50%, versus 100% English exposure). MSEL: Mullen Scales of Early Learning.


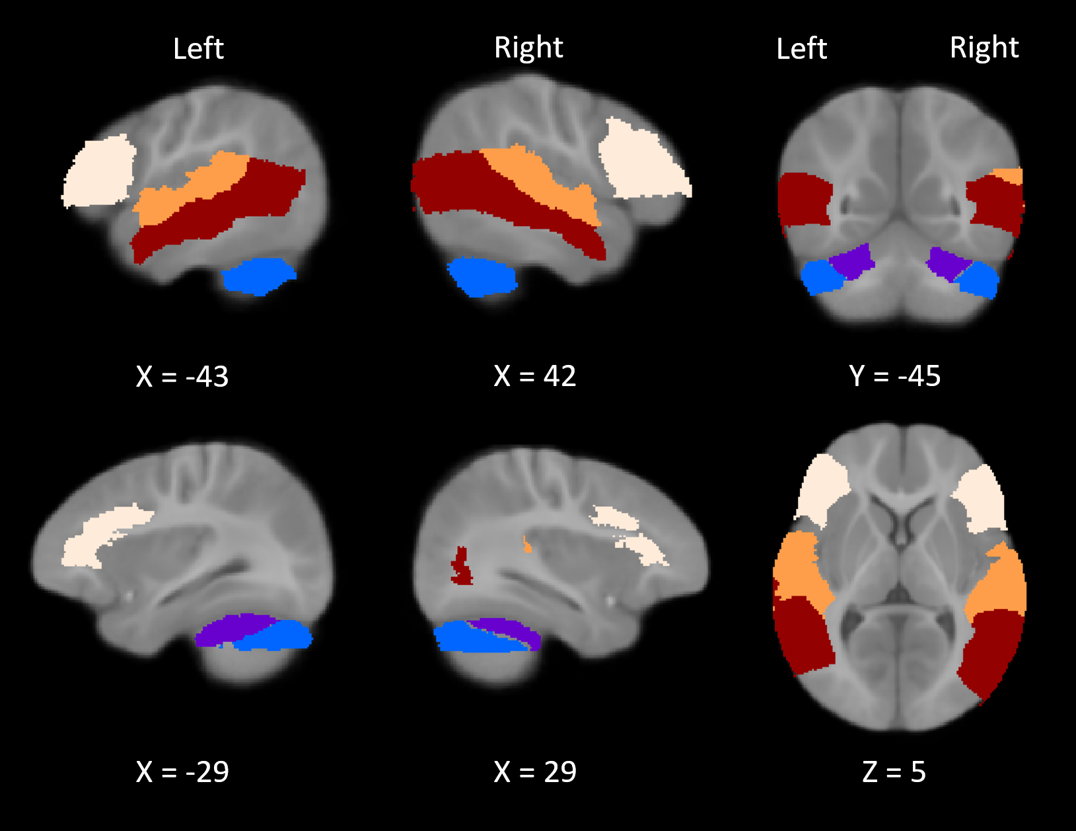


**Figure S1:** ROIs selected for the laterality analyses. Superior temporal (orange), middle temporal (red), and inferior frontal (cream) gyri, as well as the crus I (blue) and lobule VI (purple) of the cerebellum.
